# A Switch from a Gradient to a Threshold Mode in the Regulation of a Transcriptional Cascade Promotes Robust Execution of Meiosis in Budding Yeast

**DOI:** 10.1371/journal.pone.0011005

**Published:** 2010-06-08

**Authors:** Vyacheslav Gurevich, Yona Kassir

**Affiliations:** Department of Biology, Technion-Israel Institute of Technology, Haifa, Israel; University of Missouri-Kansas City, United States of America

## Abstract

Tight regulation of developmental pathways is of critical importance to all organisms, and is achieved by a transcriptional cascade ensuring the coordinated expression of sets of genes. We aimed to explore whether a strong signal is required to enter and complete a developmental pathway, by using meiosis in budding yeast as a model. We demonstrate that meiosis in budding yeast is insensitive to drastic changes in the levels of its consecutive positive regulators (Ime1, Ime2, and Ndt80). Entry into DNA replication is not correlated with the time of transcription of the early genes that regulate this event. Entry into nuclear division is directly regulated by the time of transcription of the middle genes, as premature transcription of their activator *NDT80*, leads to a premature entry into the first meiotic division, and loss of coordination between DNA replication and nuclear division. We demonstrate that Cdk1/Cln3 functions as a negative regulator of Ime2, and that ectopic expression of Cln3 delays entry into nuclear division as well as *NDT80* transcription. Because Ime2 functions as a positive regulator for premeiotic DNA replication and *NDT80* transcription, as well as a negative regulator of Cdk/Cln, we suggest that a double negative feedback loop between Ime2 and Cdk1/Cln3 promotes a bistable switch from the cell cycle to meiosis. Moreover, our results suggest a regulatory mode switch that ensures robust meiosis as the transcription of the early meiosis-specific genes responds in a graded mode to Ime1 levels, whereas that of the middle and late genes as well as initiation of DNA replication, are regulated in a threshold mode.

## Introduction

Precise and complex regulation is required for entering a developmental pathway at the correct time and in the appropriate cell type. Deviations from this regulation may lead to genome instability, causing either cell death or the formation of tumor cells [1]. Inducing the correct set of genes in a coordinated manner is a key for developmental pathway regulation and is often achieved through a transcriptional regulatory cascade [2], [3], [4]. The master activator initiating the cascade is usually controlled by multiple input signals, each with a small impact. It is the combinational nature of the induction of the master activator that ensures the correct spatial and temporal activity of the developmental pathway [2], [3], [4].

Transmission of a strong and short-lived signal by the master activator is assumed to be critical for the successful completion of a developmental pathway [1]. Studies in mice [5] and yeast meiosis [6] have demonstrated the importance of a short-lived signal for efficient entry into a developmental pathway. However, whether a *strong* signal is indeed essential for efficient entry into and completion of a developmental pathway and, if not, how cells cope with premature, delayed, reduced, or increased signals, remains a fundamental, unsolved question.

**Figure 1:**
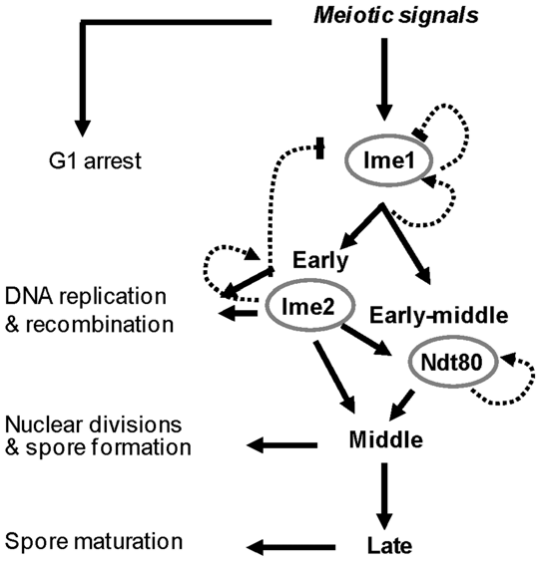
A schematic model illustrating the transcriptional cascade that governs meiosis in *S. cerevisiae*.

Our model system is meiosis in *Saccharomyces cerevisiae*, in which a transcriptional cascade governs the initiation and progression through meiosis (Figure 1 and [7], [8]). This cascade consists of a master activator, Ime1, which is essential for the transcription of the early meiosis-specific genes. The transcription of the middle genes depends on Ime2, a kinase that belongs to the early genes, and Ndt80, a transcriptional activator that belongs to the early middle genes. The transcription of the late genes is indirectly dependent on Ime1, Ime2 and Ndt80 [9]. The early genes encode proteins involved in DNA replication, synapsis of homologs and meiotic recombination, whereas the middle genes encode proteins required for nuclear divisions and spore formation [10].

We envision two modes by which a transcriptional cascade is regulated. (*i*) Transcription of a network of genes (B) responds to a gradient (graded) effect produced by a master activator (A), ie, the quantity of A directly affects the quantity of B, and (*ii*) transcription of the B genes requires a threshold level/activity of A. The first hypothesis is supported by a discrete computational model that faithfully describes the relations between a master transcriptional activator, (*IME1*) in budding yeast, and the transcription of *IME2*, an early meiosis-specific gene representing the B genes [11]. The second hypothesis is supported by two observations. First, a study of the meiosis transcriptional cascade in *S. cerevisiae* using time-lapse fluorescence microscopy of single cells demonstrated high variability in the duration of the first stage in the cascade (corresponding to the induction time of the early meiosis-specific genes) and in the level of Ime1 [12]. These results suggest that the induction of meiosis depends on a threshold level of Ime1. Second, pattern formation in Drosophila depends on a “buffered protein” that causes a gradient of a morphogen to regulate transcription in a threshold mode [13].

In this report we demonstrate that meiosis is not sensitive (robust) to increased or decreased transcript abundance of *IME1*, or to the level of expression of meiosis-specific genes. Transcription of the early meiosis-specific genes responds in a graded mode to the Ime1 levels. However, entry into premeiotic DNA replication is regulated in a threshold mode, namely, a decrease in the level of Ime1 and the early genes leads to a delayed entry into premeiotic S phase. The transcription of *NDT80*, the middle and late meiosis-specific genes are regulated in a threshold manner. We show that the time of transcription of *NDT80* and the middle genes determines the time cells enter nuclear divisions, as premature transcription of *NDT80* resulted in premature nuclear division and a reduction in asci formation. Correlation between premeiotic DNA replication and nuclear division is regulated by an early meiosis-specific gene whose activity is regulated in a threshold mode. We suggest that Ime2, which controls both initiation of premeiotic DNA replication and the transcription of *NDT80*, serves as the regulator that switches the graded mode of transcription of the early genes to a threshold mode for regulating entry into DNA replication and the transcription of the middle genes. We demonstrate that Cdk functions as a negative regulator of Ime2, by showing that point mutations of three serine/threonine residues in putative Cdk1 phosphorylation sites of Ime2 result in premature entry into premeiotic DNA replication. Accordingly, ectopic expression of the G1 cyclin *CLN3* has the opposing effect, namely delaying entry into DNA replication and *NDT80* transcription. Because Ime2 functions as a negative regulator of Cdk1/G1 cyclins, we suggest that a double negative feedback loop between Ime2 and Cdk1/Cln3 is responsible for the switch from a graded to a threshold mode of regulation. This switch provides the required coordination between DNA replication and nuclear division, ensuring robust meiosis.

## Results

### Meiosis is characterized by a transcriptional cascade

**Figure 2:**
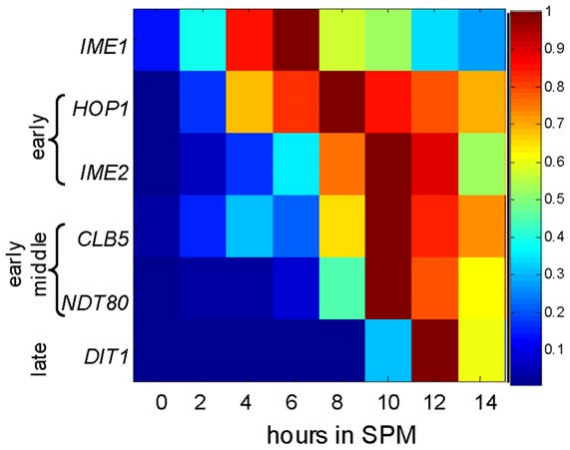
Normalized level of transcription of representatives of meiosis-specific genes. Wild type diploid cells (Y1631) were shifted to meiotic conditions (sporulation medium, SPM), and RNA was isolated at the indicated hours. Transcripts levels were measured by qPCR. The relative level of RNA in comparison to RNA levels of *ACT1* was normalized for the maximal level of expression of each gene, and set to 1. The experiment was repeated 3 times and a representative result is shown.

**Figure 3:**
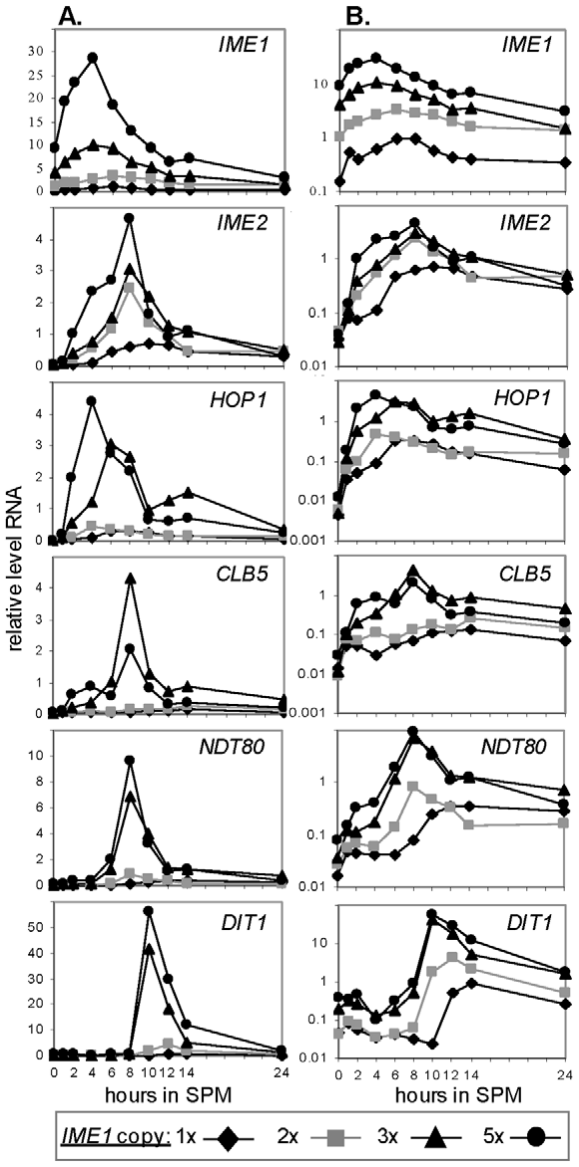
Increase in *IME1* copy number results in an increase in the level of expression of *IME1* and all meiosis-specific genes. Isogenic diploids carrying 1 (Y1639, diamonds), 2 (Y1631, squares), 3 (Y1663, triangles) and 5 (Y1662, circles) copies of *IME1* were shifted to meiotic conditions (SPM). Samples were taken at the indicated times to isolate RNA and estimate transcript levels by qPCR. **A.** The RNA level of the indicated gene relative to RNA levels of *ACT1*. **B.** The relative RNA level is drawn in a log scale. The experiment was repeated 3 times and a representative result is shown.

We used qPCR to determine the pattern of expression of several representatives of genes induced in meiosis at specific times. *IME1* was the first gene induced (Figure 2 and 3A), followed by the simultaneous induction of the early genes, *IME2* and *HOP1* (Figure 2 and 3A). The transcription of the early-middle gene *NDT80* and the late gene *DIT1* followed sequentially (Figure 2 and 3A). *CLB5* was previously designated as a middle gene whose transcription depends on Ndt80 [7], [14]. However, the use of the sensitive qPCR assay revealed that its transcription was also partially increased prior to the increase in the level of *NDT80* RNA (Figure 3A). Moreover, its initial induction was also regulated by Ime1, as its level was correlated with that of Ime1 (Figure 3A). Thus, these results are in agreement with those of Raithatha and Stuart that demonstrated a meiosis-specific Ndt80-independent transcription of *CLB5* in a deletion analysis of the *CLB5* promoter [15]. Finally, our findings demonstrate that the representative meiosis-specific genes were induced in a sequential and transient manner.

### Meiosis is robust to decreased or increased levels of *IME1* RNA

**Figure 4:**
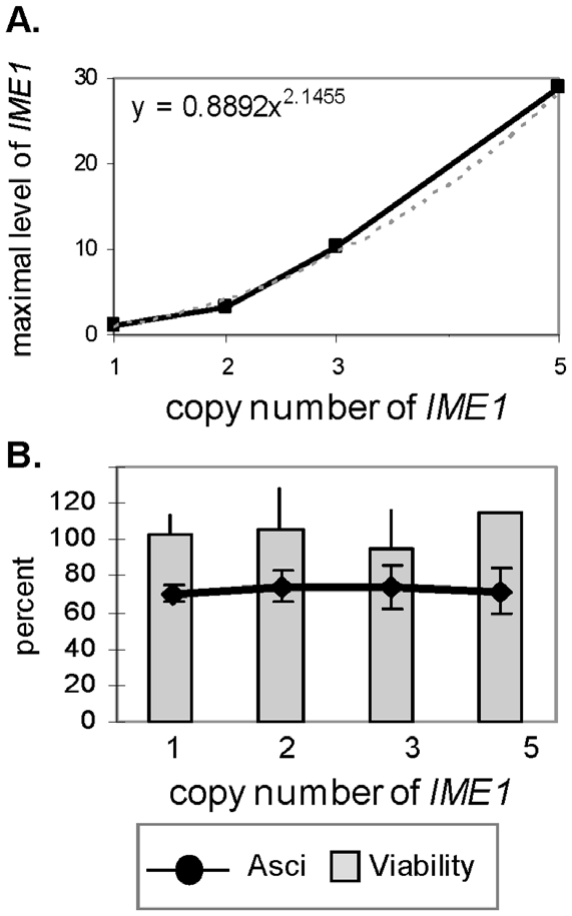
Meiosis is insensitive to the level of *IME1* mRNA. Isogenic diploids carrying different copy numbers of *IME1* were shifted to meiotic inducing conditions. **A.** The correlation between *IME1* copy number and its maximal level of expression. The maximal level of *IME1* RNA (from Fig. 3A) in comparison to the copy number of the *IME1* gene was drawn. The fitted trend line (dotted) and its formula are given. **B.** Efficiency of meiosis. The percentage of asci (line) and the number of cells that can form colonies (viability, column) at 48 hours in SPM. The results are the averages of three independent transformants and a count of ≥200 cells. Error bars represent standard deviations.

We examined whether the pattern of *IME1* expression is essential for efficient meiosis by studying isogenic strains carrying 1, 2, 3 or 5 copies of *IME1*. As expected, the increase in *IME1* copy number resulted in a substantial increase in the level of *IME1* RNA throughout the meiotic pathway (Figure 3A). The correlation between *IME1* copy number and the maximal level of *IME1* RNA was found to be non-linear (Figure 4A); Rather, it fitted a quadratic trend (ie, a polynomial of order 2) probably resulting from its positive autoregulation [9], [11], [16].

Modulation in *IME1* copy number had no deleterious effect on sporulation efficiency, as determined by the similar viability and percentage of asci formation (Figure 4B). Our results indicate that meiosis is insensitive to changes in the transcript level of its master regulator, Ime1.

### Transcription of the early meiosis-specific genes responded to Ime1 levels in a graded mode, whereas transcription of the early-middle and late genes responded in a threshold mode

Two possible scenarios can explain why the level of *IME1* RNA had no effect on meiosis efficiency: (*i*) the level of Ime1 protein might buffer the level of its RNA, creating a threshold effect, and (*ii*) the activity of an additional positive regulator might buffer Ime1 levels. The first hypothesis predicts that the expression levels of meiosis-specific genes will be insensitive to the level of *IME1* RNA. Therefore, we measured the transcript levels of representatives of meiosis-specific genes (Figure 3A). We found that an increase in the level of *IME1* RNA led to a subsequent increase in the levels of *IME2*, *HOP1* (early genes), *NDT80* (an early-middle gene), *CLB5* (an early-middle gene) and *DIT1* (an early-late gene), contrary to the first hypothesis. The second hypothesis predicts that only the early genes, which are directly regulated by Ime1, will respond in a graded mode to Ime1 levels, whereas the transcription of the middle genes will not. To examine this hypothesis, we determined how Ime1 affects the transcription time of these genes by plotting the level of transcription levels on a log scale (Figure 3B). The response of *IME2* and *HOP1* to the increasing levels of Ime1 was in a graded mode, responding to any level of Ime1 (ie, increased levels of *IME1* RNA due to an increased copy number of the *IME1* gene, resulted in a gradual increase in the transcript levels of *IME2* and *HOP1*, Figure 3B). Notably, the response of *IME2* and *HOP1* to Ime1 levels also showed a slight threshold effect. For example, after 6 hours in sporulation medium (SPM) the RNA level of *IME1* in cells containing 5 copies of *IME1* was 5.7-fold higher than that observed in the strain with 2 copies of *IME1*, whereas RNA levels of *IME2* and *HOP1*, were 2.3-, and 7.2–fold higher, respectively. We suggest that this “threshold” effect is due to the positive regulation of the early genes transcription by Ime2 (Fig. 1 and [9]). In contrast, a true threshold response was observed for the late gene *DIT1*: a decrease in *IME1* copy number resulted in its delayed transcription, whereas an increase in *IME1* copy number resulted in its advanced transcription (Figure 3B). Furthermore, the early-middle genes, *NDT80* and *CLB5*, showed a mixed response to the changes in Ime1 levels. At early meiotic times they showed a graded response, and at later meiotic times a threshold effect (delayed or advanced time of transcription). We suggest that this observation reflects a mixed mode of regulation for *NTD80* and *CLB5* by both Ime1 and Ndt80 (Figure 3A and [15], [17]).

The abovementioned results suggest that an early meiosis-specific gene which functions as a positive regulator for the transcription of *NDT80* and the middle genes acts as a buffer that switches a graded response to a threshold response.

### Coordination between the transcription of meiosis-specific genes and meiotic events

**Figure 5:**
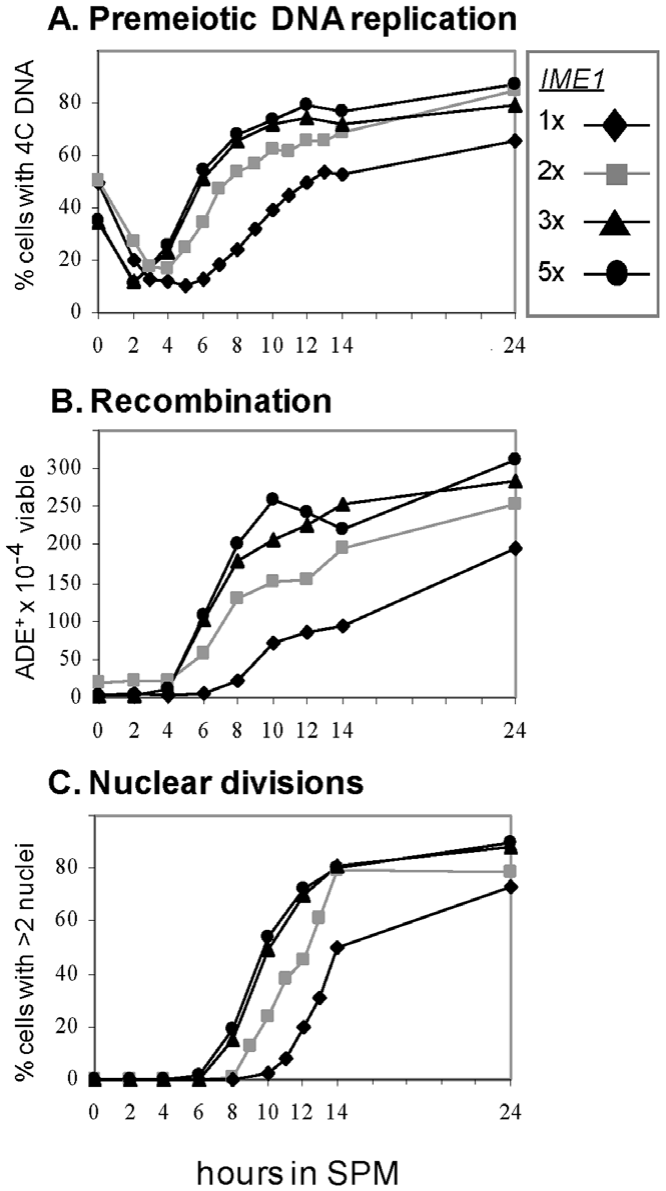
The level of *IME1* affects the time cells initiate premeiotic DNA replication, recombination and nuclear division. Isogenic diploids carrying different copy numbers of *IME1* were shifted to meiotic conditions. Samples were taken at the indicated times for FACS analysis and to calculate the percentage of cells with 4C DNA content (A), to plate on minus adenine medium and YEPD to measure the level of intragenic recombination at the *ADE2* locus (B), and to stain with DAPI to count the percentage of cells with more than 2 nuclei (C).

We examined the effect of *IME1* copy number on various meiotic events in order to elucidate how cells cope with drastic changes in the levels of positive regulators. Premeiotic DNA replication, commitment to meiotic recombination, and nuclear divisions were delayed in cells carrying a single copy of *IME1* (compared to cells with 2 copies of *IME1*, Figure 5), whereas the transcription of the early genes *IME2* and *HOP1* was not delayed (Figure 3). Moreover, these events were induced at an earlier time in cells carrying 3 or 5 copies of *IME1* (Figure 5). These results indicate that initiation of early meiotic events (ie, DNA replication and commitment to meiotic recombination) are not correlated with the time of transcription of the early meiosis-specific genes that encode proteins required for these events. Conversely, entry into nuclear division is correlated with the time of transcription of *NDT80*.

### Premature transcription of middle genes is deleterious to meiosis

**Figure 6:**
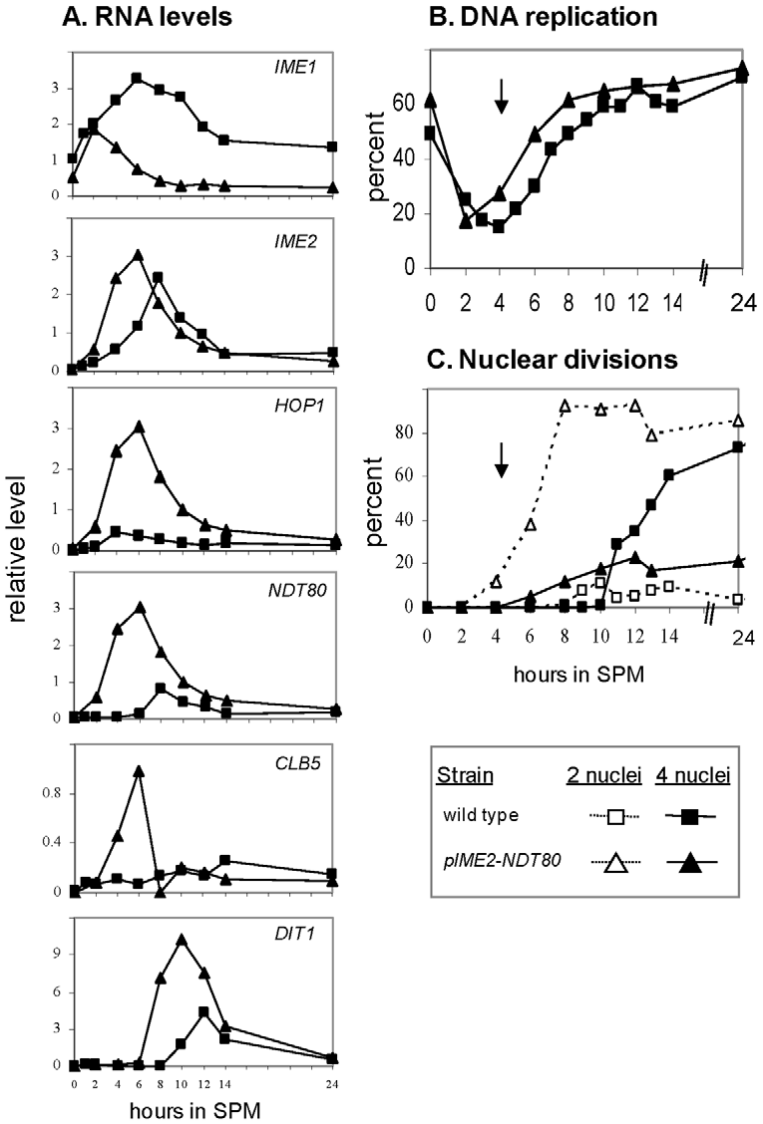
Premature transcription of *NDT80* results in a premature entry into nuclear division, and consequently a defective meiosis. Isogenic *NDT80*/*NDT80* (Y1631, squares) and *ndt80*Δ*C'::IME2p-NDT80-TRP/ndt80*Δ*C'::IME2p-NDT80-TRP1* (Y1764, triangles) cells were shifted to meiotic conditions (SPM), and at the indicated hours samples were taken to extract RNA (A), to process for FACS analysis and calculate the percentage of cells with 4C DNA content (B), and to stain with DAPI to count the percentage of cells with 2 (open squares and triangles, dashed lines) and 4 nuclei (filled squares and triangles) (C). Arrows mark initiation time for DNA replication and nuclear division.

We hypothesized that the delay in nuclear division observed in cells carrying a single copy of *IME1* was due to the delay in the transcription of the middle genes that encode proteins required for nuclear division. This hypothesis predicts that premature transcription of the middle genes will cause premature entry into the first meiotic division (MI), loss of coordination with DNA synthesis and consequently a defect in meiosis. We achieved premature expression of the middle genes (*NDT80*, *CLB5*, and *DIT1*) by generating a mutant expressing Ndt80, their transcriptional activator, from the *IME2* promoter (Figure 6A). Premature transcription of *NDT80* resulted in reduced and constricted expression levels of *IME1*, *IME2*, and *HOP1* (Figure 6A). These results imply that either Ndt80 has multiple roles, functioning both as a positive and a negative regulator, or that the negative effects of Ndt80 are mediated through its positive effect on the transcription of the middle meiosis-specific genes. Moreover, premature transcription of *NDT80* induced the transcription of *IME2* (Figure 6A), a result supported by studies demonstrating that Ndt80 is a positive transcriptional activator of *IME2*, and the existence of an Ndt80 consensus site within 600 bp upstream of *IME2* [18].

Premature transcription of *NDT80* advanced entry into meiotic S by one hour compared to the wild type (wt) strain (Figure 6B). We suggest that the advanced entry into meiotic S was due to the increased transcript levels of *CLB5* (Figure 6A), which is required for initiation of premeiotic DNA replication [19], [20]. The major effect observed was advancing the time cells entered MI (MI was initiated at the same time cells initiated DNA replication, Figure 6C). Moreover, the frequency of cells completing two divisions was very low and most cells showed only one nuclear division (Figure 6C). The mutant strain showed a reduction in asci formation (11.7% vs 83.8% in the wt strain). These results combined, demonstrate that time of transcription of *NDT80* and the middle genes is pivotal for efficient meiosis, as predicted by our hypothesis.

### Ime2 is the regulator that switches the mode of response to Ime1 from graded to threshold

The transcription of the early meiosis-specific genes was not delayed in cells carrying a single copy of *IME1* (Figure 3B), suggesting that the activity levels of protein(s) controlling the time cells enter meiotic DNA replication may be regulated in a threshold mode, similarly to the transcriptional regulation of *NDT80*. We hypothesized that Ime2 may be the “threshold factor” that buffers Ime1 levels, and whose activity coordinates DNA replication, *NDT80* transcription, and consequently nuclear division, as Ime2 is required for timely entry into premeiotic DNA replication and nuclear divisions, as well as for *NDT80* transcription [9], [21], [22], [23]. Since the predicted amino acid sequence of Ime2 includes three potential Cdk1 phosphorylation sites within its regulatory region (S/T-P-X-R/K at T302, and two non-perfect S/T-P sites, at T202 and S252, [9]), and since Ime2 activity depends on phosphorylation of Y244 by Cak1 [24], [25], we hypothesized that Ime2 phosphorylation by Cdk1 on T302 (as well as on T202 and S252) might hinder Cak1 ability to phosphorylate Ime2, causing a threshold effect to Ime2 activity. This hypothesis predicts that threonine to alanine mutations at these sites might promote premature phosphorylation by Cak1, and consequently premature activation of Ime2. Therefore, we constructed the *ime2-T202A,S252A,T303A* allele (designated *ime2-3SA*) by site-directed mutagenesis, and examined the ability of a diploid strain expressing the Ime2-3SA allele to promote meiosis.

**Figure 7:**
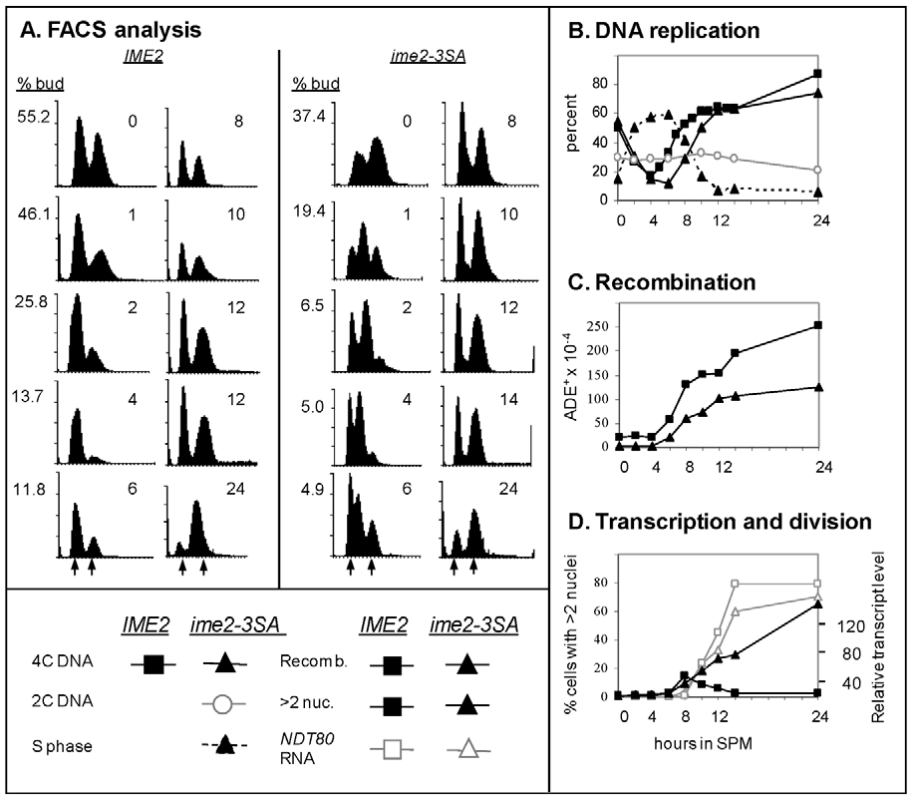
The *ime2-3SA* mutation resulted in premature entry into premeiotic DNA replication. Isogenic *IME2*/*IME2* (Y1631, squares) and *ime2-3SA/ime2-3SA* (Y1740, triangles or circles) cells were shifted to meiotic conditions (SPM). At the indicated hours samples were taken to process for FACS analysis, to count the number of unbudded cells, to plate on minus adenine and YEPD plates, to isolate RNA, and to stain with DAPI. **A.** FACS and budding index. The percentage of budded cells is given at the left side of each FACS. **B.** DNA replication. The percentage of *ime2-3SA/ime2-3SA* cells with 2C DNA content (open circles, grey line), in S phase (triangle, dashed line), or with 4C DNA content (triangle, line) was calculated. The percentage of wt cells with 4C DNA content (square, line) was calculated **C.** Commitment to intragenic recombination at the *ADE2* locus. **D.** The percentage of wild type (square) and mutant cells with more than 2 nuclei (grey lines). The relative level of *NDT80* RNA in comparison to RNA levels of *ACT1* is shown (black line). The experiment was repeated 3 times and a representative result is shown.

In wt diploids, nitrogen depletion caused accumulation of cells in G1 (determined by an increase in unbudded cells with 2C DNA content, Figure 7, A left panel and D), after which cells initiated DNA replication (after approximately 5 hours in SPM, Figures 7, A and D). Notably, bud emergence was not deleteriously affected by the *ime2-3SA* allele; Conversely, arrest as unbudded cells was faster and more efficient in cells carrying the *ime2-3SA* allele than in wt cells (Figure 7A, and see discussion). However, cells carrying the *ime2-3SA* allele initiated meiotic S prematurely; a third peak with an intermediate level of DNA content appeared after only 1 hour in SPM (Figures 7, A and B). Furthermore, the level of cells with 4C DNA content decreased in the first 6 hours in SPM, implying that cells completed the mitotic cycle, whereas the level of cells with 2C DNA content did not increase, implying that the cells did not accumulate in G1, and immediately entered the premeiotic S phase (Figure 7B). These results suggest that the Ime2-3SA protein was activated prematurely and promoted initiation of DNA replication in the mutant strain; however, the lack of accumulation of cells with 4C DNA content (Figure 7B) suggests that an additional factor (perhaps a Cdk) is required for efficient progression through DNA replication, and its absence during the premature initiation of DNA replication prevents premature completion of DNA replication.

The *ime2-3SA* allele also affected meiotic recombination, as commitment to intragenic recombination at the *ADE2* locus was delayed, and its maximal level was reduced compared to the wt strain (Figure 7C). Since *NDT80* transcription depends on the recombination checkpoint [26], we expected that the delay and reduction in meiotic recombination would impact the time of *NDT80* transcription in the mutant strain; indeed, *NDT80* transcription in the mutant strain was delayed relative to the premature entry into S (initiated at the same time as that observed in wt, Figure 7D). Furthermore, *NDT80* transcription was non-transient.

In agreement with the transcript analysis, DAPI staining revealed that MI was initiated at the same time in the wt and *ime2-3SA/ime2-3SA* strains (Figure 7D). However, in the mutant strain 27.4% of the cells remained arrested with a single nucleus, compared to only 11.2% in the wt strain. In summary, the multiple phenotypes observed suggest that *ime2-3SA* is a constitutive active allele.

### The activity of Ime2 is negatively regulated by Cdk1/Cln3

**Figure 8:**
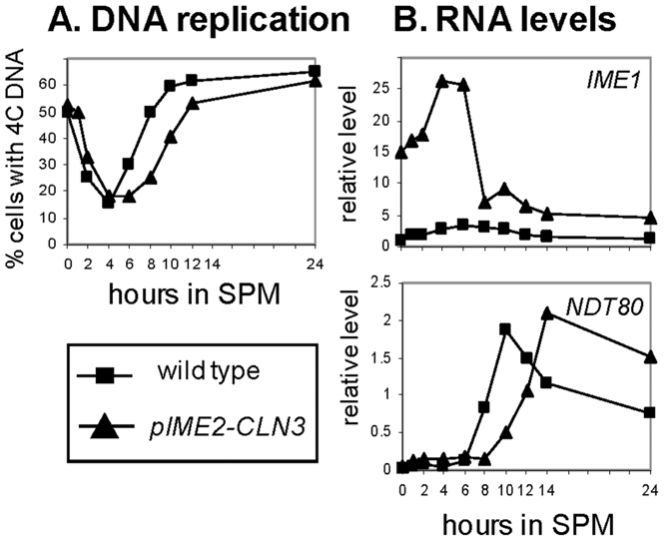
Ectopic overexpresion of Cln3 increases the transcription of *IME1*, but delays initiation of premeiotic DNA replication and *NDT80* transcription. A wt strain (Y1631) carrying *pIME2-CLN3* on a 2μ plasmid (YEp3212, triangles) or the vector plasmid (squares) were shifted to meiotic conditions. At the indicated hours samples were taken to process for FACS analysis and calculate the percentage of cells with 4C DNA content (A), and to isolate RNA and determine the relative transcripts levels of *IME1* and *NDT80* by qPCR (B).

The abovementioned results suggest that Cdk1 inhibits Ime2 activity. This hypothesis predicts that increased activity of Cdk1 at early meiotic times will delay Ime2 activity. Therefore, we increased the activity of Cdk1 at early meiotic times, by expressing the G1 cyclin *CLN3* from the *IME2* promoter. We assumed that ectopic expression of *CLN3* will increase the activity of Cdk1, because Cdk1 level is constitutive throughout the meiotic pathway [27]. We chose *IME2* and not *IME1* as the promoter to bypass the negative effect of Cdk1/G1-cyclins on the transcription of *IME1* [28]. *MAT*a/*MAT*α diploids carrying *pIME2-CLN3* on a 2μ plasmid were shifted to meiotic conditions, and transcripts levels of *IME1* and *NDT80* as well as level of DNA replication were monitored over time. Ectopic expression of *CLN3* caused a 2 hours delay in entering premeiotic DNA replication (Figure 8A), similar to the delay observed in cells carrying a single copy of *IME1* (Figure 5A). This delay was not the result of reduced *IME1* expression because conversely *IME1* expression was substantially increased (Figure 8B). In addition, ectopic expression of *CLN3* delayed *NDT80* transcription compared to wt (Figure 8B). The observed delayed entry into premeiotic S and the effects on *IME1* and *NDT80* transcription support our hypothesis that Cdk1/Cln3 inhibits Ime2 activity, as Ime2 functions as a negative regulator of *IME1* transcription and as a positive regulator of *NDT80* transcription [9]. Finally, ectopic expression of *CLN3* was deleterious, as the level of asci was 49% in comparison to about 75% in the wt strain, and the level of monads and dyads within the asci was 35% compared to 3% in the wt strain.

## Discussion

### The role of positive autoregulation in establishing robust meiosis

**Figure 9:**
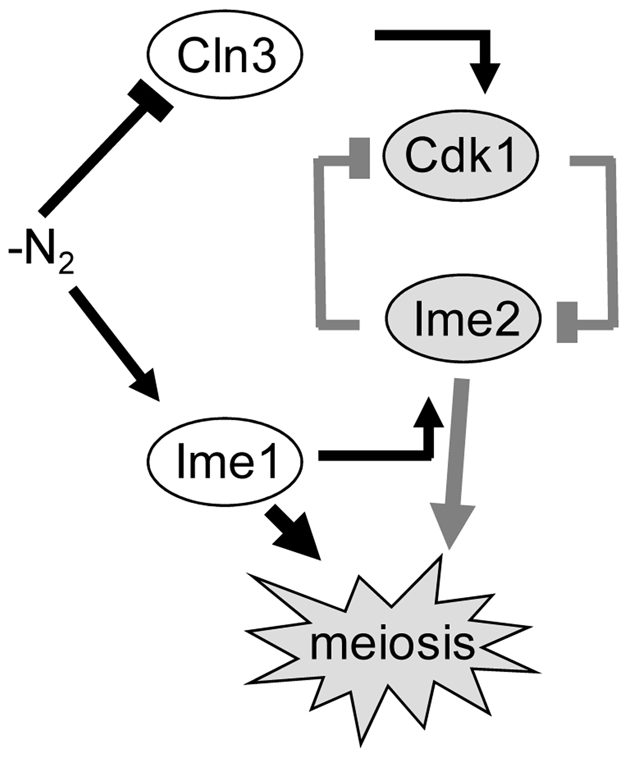
A schematic model illustrating the double negative feedback loop between Ime2 and Cdk1. Black arrows and lines illustrate graded type of regulation; grey lines and arrows represent a threshold mode of regulation.

Feedback regulation is one of the major characteristics of transcriptional activators that control entry into developmental pathways (for specific examples see [1]). In budding yeast, the transcription of the positive regulators of meiosis, *IME1*, *IME2* and *NDT80*, is subject to positive autoregulation [(Figure 1A and [9]). It is usually assumed that transcript abundance of such regulators is tightly regulated, and essential for the correct response of their target genes [1]. Therefore, we hypothesized that meiosis would be sensitive to radical changes in the levels of such positive regulators. However, our findings do not support this hypothesis because drastic changes in *IME1* levels had no deleterious effects on the efficiency of meiosis (Figures 3A, and 4B). The transcription of multiple positive regulators have been demonstrated to be negatively regulated eg, *IME1* by Cdk1/Cln and Sok2 [16], [28], *IME2* by the ATP-dependent chromatin-remodeling Isw2 factor [29], as well as by deacetylation of histones H3 and H4 [30], and *NDT80* by histone deacetylase complexes [7], [22], [31], [32], [33]. We suggest that positive autoregulation is required to overcome the initial negative regulation on the transcription and/or function of these regulators.

### Negative feedback regulation ensures a transient transcription of *IME1*

The transcription of *IME1* is transient in wt cells and non-transient in *ime2*Δ/*ime2*Δ cells [9] suggesting that Ime2 either directly or indirectly represses *IME1* transcription. Moreover, phosphorylation of Ime1 by Ime2 tags it for degradation [34]. Our data support these findings as inhibition of Ime2 function by ectopic expression of the G1 cyclin, Cln3, resulted in a substantial increase in the level of *IME1* RNA (Figure 8B). Premature transcription of *NDT80* led to reduced and constricted levels of *IME1* expression (Figure 6A), whereas its delayed transcription resulted in an increase in *IME1* transcription (Figure 8B). These results are in agreement with a prior report showing that *NDT80* deletion resulted in a non-transient transcription of *IME1* [35]. As Ime2 is required for *NDT80* transcription [36], our results suggest that the effect of Ime2 is also mediated through Ndt80. We propose that this negative feedback loop is mediated by Cdk1, as Ndt80 regulates the transcription of the B-type cyclins [7] that activate Cdk1 (Cdk1 level is constitutive in both the mitotic and meiotic cycles [27]). Phosphorylation of Ime1 by Cdk1/Cln3 sequesters it from the nucleus [28], thereby preventing positive autoregulation of Ime1. In addition, independent of Ime1, Cdk1 functions as a negative regulator for *IME1* transcription (V. Gurevich and Y. Kassir unpublished data). We further propose that the reduced levels of *IME1* caused by *NDT80* premature expression led to a decrease in the transcription of the early genes *HOP1* and *IME2* (Figure 6A).

### Robust meiosis depends on the time of *NDT80* transcription

Our findings demonstrate that meiosis in budding yeast is insensitive to a large range of *IME1* transcript abundance (Figure 4C). Notably, meiosis in budding yeast has been previously reported to be sensitive to constitutive high expression levels of Ime1, leading to a decrease in the percentage of asci and an increase in both the level of non-disjunction and dyads [6]. Our results demonstrate that robust meiosis depends on the sequential transcription of the various clusters of genes (ie, early vs. middle meiosis-specific genes). Premature transcription of *NDT80* resulted in premature transcription of the middle genes, as well as premature entry into nuclear division (Figures 6, A and C). Nuclear division is also regulated by the pachytene checkpoint which monitors defects in meiotic recombination and synapsis of homologs [26]. This checkpoint arrest is achieved by two parallel mechanisms, repressing *NDT80* transcription and inhibition of Cdk1/B-type cyclins activity by Swe1 [37]. Premature transcription of *NDT80* promoted concomitant entry into DNA replication and MI (Figure 6), suggesting that Swe1 was not active in cells expressing Ndt80 from the *IME2* promoter and implying that the Swe1-activating signal is switched on only after the initiation and/or completion of DNA replication. In accord, at early meiotic times the level of Swe1 is low, whereas during pachytene its level is transiently increased [37]. As premature expression of *NDT80* resulted in a significant reduction in the level of cells that entered MII, (most cells accumulated with 2 nuclei, Figure 6C), we suggest that MII inhibition was due to inhibition of Cdk1 activity, most probably due to Swe1 activation.

### Coordination between premeiotic DNA replication and nuclear division

We hypothesized that the coordination between meiotic S and nuclear division is accomplished by the cascade nature of the transcription of the early and middle meiosis-specific genes. The following observations support this hypothesis: (*i*) premature transcription of the middle meiosis-specific genes disrupts this coordination causing premature entry into MI (Figure 6), and (*ii*) the pachytene checkpoint, which is activated upon defects in meiotic recombination and synapsis of homologs, inhibits *NDT80* transcription, and consequently the middle genes [7], [35]. However, our hypothesis could not explain the observation that transcription of the early genes was induced in a graded mode (in cells carrying various copy numbers of the *IME1* gene), whereas premeiotic DNA replication was regulated in a threshold mode (ie, delayed in cells carrying one copy of *IME1* and advanced in cells carrying 3 or 5 copies of *IME1* compared to cells carrying 2 copies of *IME1*, Figures 3B and 5). Since *NDT80* transcription and MI entry is concomitantly delayed, we propose that the coordination between meiotic S and nuclear division depends on a switch from a gradient mode in the transcription of early meiosis genes to a threshold mode for both initiation of meiotic S and *NDT80* transcription.

### Ime2 serves as the buffer responsible for the switch from a graded to a threshold mode of response

We propose that the switch from a graded to a threshold mode in regulation of premeiotic DNA replication and the transcription of *NDT80* is mediated through Ime2. This hypothesis is based on the following observations: (*i*) deletion or a kinase-dead mutation in *IME2* results in a delayed entry into meiotic S and nuclear division [21], [27], (*ii*) in cells expressing the *ime2-3SA* allele meiotic S is induced prematurely (Figure 7A), and (*iii*) Ime2 is essential for the transcription of *NDT80* [36]. The transcription of *IME2* is regulated in a graded mode (Figure 3), but we propose that its activity is regulated in a threshold mode. Such a switch from a graded to a threshold mode is theoretically possible, based on mathematical analyses [2], [38], [39], [40], [41]. We suggest that the mechanism for the switch in Ime2 activity is a double-negative feedback loop between Cdk1/Cln3 and Ime2 (Figure 9). The following results support Cdk1 as an Ime2 inhibitor: (*i*) mutations in three putative Cdk phosphorylation residues in Ime2 (*ime2-3SA*) caused premature entry into premeiotic DNA replication (Figure 7A), and (*ii*) ectopic over expression of the G1 cyclin Cln3 caused an increase in the level of *IME1* RNA as well as a delay in entering premeiotic S phase and the transcription of *NDT80* (Figure 8). The following lines of evidence support Ime2 as a negative regulator of Cdk1/G1 cyclins function which is required for bud emergence [42]: (*i*) Ectopic overexpression of Ime2 is associated with accumulation of unbudded cells ([43] and M. Szwarcwort-Cohen and Y. Kassir unpublished data), (*ii*) ectopic expression of either mouse Cdk1 or human Cdk2 prevented nitrogen-depletion induced G1 arrest only in *ime2*Δ diploids but not in wt strain [44], suggesting that Ime2 contributes to the G1 arrest, and (*iii*) in cells carrying the constitutive active *ime2-3SA* allele, the level of budded cells is reduced in comparison to the wt strain, and upon nitrogen depletion the mutant cells arrest at G1 more efficiently (Figure 7). Taken together, the results suggest that these two kinases, Ime2 and Cdk1/Cln interact in a double-negative loop (Figure 9). Upon nitrogen depletion the activity of Cdk1 is reduced in a graded mode due to the absence of cyclins [45], [46]. Nitrogen depletion also leads to a graded increase in the level of Ime1, and consequently Ime2. These two opposing effects may, at a certain level of Cdk1/Cyclin and Ime2, constitute a bistable switch between cells exhibiting Cdk and Ime2 activity. The mathematical model of James Ferrel [2] demonstrates that such a double negative feedback loop can switch a graded response to a threshold response.

In summary, our results explain how the meiosis developmental pathway is a robust process, initiated only under the right conditions, and the way by which two different regulation modes (ie, graded and threshold) are used consecutively to induce the pathway at any level of Ime1, and to coordinate early and late meiotic events. Moreover, the proposed double negative feedback loop between the positive regulators of the cell cycle (Cdk) and meiosis (Ime2) explains the mechanism by which meiosis and the cell cycle exist as mutually exclusive developmental pathways, that is entering one pathway inhibits the concomitant entry into the second pathway.

## Materials and Methods

### Strains

Strains are listed in Table 1. Y1659 and Y1660 were constructed by transforming Y1065 with YIp2234 digested with PpuMI. qPCR analysis reveled that they carry 4 and 2 copies of *IME1*, respectively. Y1735 and Y1736 were constructed by transforming Y1064 and Y1065, respectively with YIp3045 cut BstEII. Y1760 and Y1761 were constructed by transforming Y1065 and Y1075, respectively, with YIp3078 digested with EcoRI. Because the EcoRI site is within the *NDT80* ORF, transformation resulted in the formation of a *ndt80ΔC'::IME2p-NDT80-TRP1* loci. Genotypes were confirmed by PCR.

**Table 1:** List of strains.

*Strain*	*Relevant genotype*	*Remarks and references*
Y546	*MAT*a, *ura3-52*, *trp1*Δ, *leu2-3,112*, *his4-519*, *ade2-1*	
Y1064	*MAT*a, *ura3-52*, *trp1*Δ, *leu2-3,112*, *his3::hisG*, *ade2-1*	Isogenic to Y546 [16]
Y1065	*MAT*α, *ura3-52*, *trp1*Δ, *leu2-3,112*, *his3::hisG*, *ade2-R8*	[16]
Y1075	*MAT*a, *ura3-52*, *trp1*Δ, *leu2-3,112*, *his3::hisG*, *ade2-1*, *ime1::hisG*	Isogenic to Y1064 [16]
Y1659	*MAT*α, *ura3-52*, *trp1*Δ, *his3::hisG*, *ade2-R8*, *leu2,3-112::*[*LEU2-IME1(−1439 to +1081)-ADH1t*]3×	Isogenic to Y1065
Y1660	*MAT*α, *ura3-52*, *trp1*Δ, *his3::hisG*, *ade2-R8*, *leu2,3-112::*[*LEU2-IME1(−1439 to +1081)-ADH1t*]1×	Isogenic to Y1065
Y1631	*MAT*a/*MAT*α *IME1/IME1*	Y1064×Y1065
Y1639	*MAT*a/*MAT*α *ime1::hisG/IME1*	Y1075×Y1065
Y1662	*MAT*a/*MAT*α *IME1/IME1*, *leu2,3-112/leu2,3-112::*[*LEU2-IME1(−1439 to +1081)-ADH1t*]3×	Y1064×Y1659
Y1663	*MAT*a/*MAT*α *IME1/IME1*, *leu2,3-112/leu2,3-112::*[*LEU2-IME1(−1439 to +1081)-ADH1t*]1×	Y1064×Y1660
Y1735	*MAT*a, *ime2(−23 to +2231)::IME2-T202A, S252A,T303A)-URA3*	Isogenic to Y1064
Y1736	*MAT*α, *ime2(−23 to +2231)::IME2-T202A, S252A,T303A-URA3*	Isogenic to Y1065
Y1740	*MAT*a/*MAT*α, *ime2(−23 to +2231)::IME2-T202A*, *S252A,T303A-URA3/ime2(−23 to +2231)::IME2-T202A*, *S252A,T303A-URA3*	Y1735×Y1736
Y1760	*MAT*a, *ura3-52*, *trp1*Δ, *leu2-3,112*, *ade2-1*, *his4-519*, *ndt80ΔC'::IME2p-NDT80-TRP1*	Isogenic to Y546
Y1761	*MAT*α, *ura3-52*, *leu2,3-112*, *trp1*Δ, *his3::hisG*, *ade2-R8*, *ndt80ΔC'::IME2p-NDT80-TRP1*	Isogenic to Y1065
Y1764	*MATa/MATα*, *ndt80ΔC'::IME2p-NDT80-TRP1/ndt80ΔC'::IME2p-NDT80-TRP1*	Y1760×Y1761

### Plasmids

YIp2234 carries *IME1*(−1439 to +1081)-*ADHt* on a *LEU2* vector. YIp3045 carries *IME2-T202A,S252A,T303A (−23 to +2231)* on a *URA3* vector. The plasmid was constructed by site-directed mutagenesis, and its sequence was verified. YIp3078 carries *pIME2-ndt80*(*1-1223*) on a *TRP1* vector. YEp3212 carries *pIME2-CLN3* on a *URA3* 2μ vector. A detailed description on the constructions of these plasmids is available upon request.

### Media and Genetic Techniques

Minimal acetate medium (PSP2) and sporulation medium (SPM) have been described previously [47]. Meiosis was induced as follows: cells were grown in PSP2 supplemented with the required amino acids to early exponential stage (0.8–1.2×10^7^ cells/ml), washed once with water, and resuspended in SPM. The number of cells that can form colonies (viability) was measured as described [47]. Staining with 4′-6-diamidino-2-phenylindole (DAPI) was performed as described [48]. Intragenic meiotic recombination within the *ADE2* locus was performed as described [47]. As time of meiotic events can vary between experiments done at different days, the results described herein are from a single experiment which was repeated ≥3 times (with all experiments showing the same behavior). Moreover, when different meiotic events are reported, they are all from the same experiment.

### FACS Analysis

Cells were analyzed for DNA content by FACS analysis as described previously [21], using a FACScan analyzer (BD Biosciences, San Jose, CA). The percentage of cells with 2C, 4C and intermediate levels of DNA content was calculated using the WinMDI program.

### Quantitative analysis of RNA level

RNA was extracted from 10^8^ cells by the hot acidic phenol method. Up to one microgram of total RNA was used for a reverse transcription reaction (total 20 µl) with random decamer primers and SuperScript reverse-iT™ transcriptase. The resulting cDNA was then used for real-time PCR (qPCR) analysis according to the manufacturer's instructions (ABGene, Surrey, U.K.).

### Oligonucleotides

The sequences of the oligonucleotides used in this study are available upon request.
